# Construction and validation of a new Generational Time Perspective Questionnaire

**DOI:** 10.1038/s41598-024-64185-3

**Published:** 2024-06-10

**Authors:** Celina Timoszyk-Tomczak, Piotr Próchniak

**Affiliations:** Institute of Psychology, University of Szczecin, Krakowska 69, 70-017 Szczecin, Poland

**Keywords:** Psychology, Health occupations

## Abstract

The article presents a proposal for a new diagnostic tool—the Generational Time Perspective Questionnaire. The Generational Time Perspective was defined as a cognitive-affective representation of the future in relation to the lives of a generation of people that the current generation of people will not live to see. This definition was the starting point for the construction of the Generational Time Perspective Questionnaire. The results of exploratory and confirmatory factor analyses indicate a two-factor structure of the Generational Time Perspective Questionnaire. The first factor includes items about the cognitive representation of the future in the next generations. The second factor describes negative emotions towards the problems that humanity may face in future generations. The reliability of the particular dimensions of the questionnaire is satisfactory. The Generational Time Perspective Questionnaire correlates with other constructs that diagnose different aspects of an individual’s temporality.

## Introduction

Human life takes place in time, understood not only in the physical but in the psychological sense. Physical time is a reference point for different events. In turn, psychological time is a cognitive construct that allows us to almost completely transcend the limitations of the real world^1^. Perceived time is a prerequisite for creating and implementing one’s own goals and life plans, building one’s own identity and understanding personal meaning of life^2–9^.

Mental time can be treated as an important aspect of human consciousness because it involves the processes of attention, memory, information processing, and the formation of concepts and judgements. It is purely subjective. For this reason, the passage of time depends on subjective (e.g. personality traits) and objective (e.g. situational) conditions. Psychological time also includes orientation to the basic temporal dimensions (past, present, future) with their content and direction, awareness of the pace of the passage of time, and knowledge about time and the regulatory role of time in human behaviour^10^.

Lewin^11^ was one of the first researchers interested in the issue of time perspective in psychology. To him, time perspective includes a person’s relationship with their own past, present and future. Thoughts and feelings associated with each of these dimensions can have a significant impact on current human behaviour. In the 1960s, Fraise^12^ drew attention to the importance of time perspective in the course of behaviour. According to him, not only current experiences but also those stored in our memory and expectations for the future significantly determine our actions: ‘The present therefore has several dimensions: the present of things past, the present of things present and the present of things future’^12^. Nuttin and Lens^13^ believe that the temporal perspective is a mental construct that enables a person to relate to their own past, present and future. This reference manifests itself in the ability to distance oneself from past experiences and anticipate the achievement of future goals. It is thanks to this process that it becomes possible to transcend the limitations of physical space–time^1^. Block^14^, on the other hand, believes that time perspective describes the ways in which people relate to their own past, present and future.

Contemporary research on the temporal horizon is developing in different directions^5–8,13,15–17^. Most often, researchers use the term ‘time perspective’ or ‘temporal orientation’ to describe the time horizon that includes the past, present and future^9^. As a result of the research conducted, there are new attempts to describe important aspects of time perspective. Zimbardo distinguished between a positive and negative past, a hedonistic and fatalistic present, and a future perspective^5^. In further analyses, a positive and negative future also were distinguished^18^. A balanced time perspective, which refers to the ability to effectively switch between time perspectives, allows for effective adaptation to the current situation^19^. There are also attempts to reconceptualise the time perspective by distinguishing between the state of TP (state-TP), i.e. a temporary focus on one of the time horizons, and the trait of TP (trait-TP), which is described as a relatively constant differential trait with additional attitude components^7,20^.

In addition to the ‘traditional’ dimensions of time perspective—past, present and future—some researchers distinguish the representation of time up to a person’s birth (historical past) and the time after death, defined as the transcendental time perspective^5,21^. Research into the transcendental future is a relatively new trend in psychology. Transcendental thinking about the future is associated with religion and spirituality. The main concepts of transcendental thinking about the future were presented by van Beek and Kairys^22^, referring to R. J. Lifton’s Death Transcendence Theory, E. Becker’s Terror Management Theory, and P. G. Zimbardo and J. N. Boyd’s Transcendental Future Time Perspective concept. Boyd and Zimbardo^23^ propose a transcendental time perspective^5^. The transcendental future involves the ability to believe, think, and imagine immortality. It concerns the time after a person’s death, beyond the individual’s imagination. It is linked to the hope of an eternal life. Analyses and research in this field may suggest that the future beyond the temporal is not a homogeneous area. Timoszyk-Tomczak and Bugajska^24^ suggest distinguishing between the transcendental and the transcendent future.

The transcendental nature of the future is primarily associated with faith and thinking about the time after death in terms of personal ideas, such as meeting loved ones or salvation. On the other hand, the transcendence of the future includes the personal and generational dimensions as well as the metaphysical aspect^25^. Transcendentality and transcendence are not completely separate, but they have a different specificity. They can complement each other, but they can also function relatively independently. Transcendentality may be more related to the cultural message, including attitudes towards religion. In turn, transcendence may be more related to the internalisation of values or patterns and broader spiritual development.

The above suggestions for forms or types of time perspective have referred directly to the personal time of an individual’s life, both the present and that which concerns the existence after the individual’s death. However, it seems that an individual’s time perspective may include not only cognitive representations of time related to one’s own life, but also a time perspective that goes beyond personal projections. The time perspective that goes beyond the personal ‘I’ is also playing an increasingly important regulatory role in individuals’ current behaviour and concerns the focus on the lifetime of future generations^26,27^. The simplest examples of this seem to be all kinds of activities aimed at protecting our planet from climate change caused by human activities. Phasing out fossil fuels, limiting the production of plastics, all kinds of pro-ecological activities (water saving, waste separation, etc.) are clear examples of focusing on the lives of future generations. Research shows the relationship between the time perspective, especially the future, and pro-environmental attitudes and behaviours^28^.

The basis for distinguishing a generational future is the capability of mental time travel. People, thanks to their unique form of chronesthesia consciousness, can think about the subjective time in which they live and mentally travel in it to the episodic past and future.

Mental time travel is generative, allowing us to return to what was, to anticipate what will be, and to create entirely imaginary stories^29–32^. The mental journey is multidirectional: we can move from the present to the past and the future, but also vice versa, from the past or the future to look at the present.

Humans have the unique ability to construct an alternative reality in their minds^33^. Mental travelling allows us not only to travel through the time of our personal lives but also to go beyond our own existence. People can travel to the time before their own birth and after their death^34^. An attempt to combine the concepts of mental journeys is also presented by Stocker^35^, who talks about the time machine in our mind. Travelling through time in our own minds allows us to see the passing of generations and imagine the conditions and problems they will face.

Mental travelling also allows the creation of ideas about who one might become in the future (the future self). The future self encompasses the ideal selves that individuals hope to become, other selves that individuals could become, and selves that individuals are afraid of becoming. The prospective self may be employed as a map, indicating the manner in which individuals may progress from their existing position to the state they have imagined^36^.

It is evident that embarking on a mental journey beyond one's immediate surroundings inevitably entails a certain degree of uncertainty. Whilst uncertainty is capable of evoking a range of emotional states, including feelings of anxiety and fear, it can also inspire feelings of hope and optimism. However, these emotional reactions may vary considerably depending on an individual’s personal differences in how people handle uncertainty^37^.

The capacity to mentally traverse the future can extend beyond the scope of an individual’s life, encompassing the perspective that future generations may not experience the world in the way we currently do. Therefore, we propose a new form of time perspective—a generational time perspective. The generational time perspective is a cognitive-affective representation of the future relating to the life of a generation of people that the current generation of people will not live to see. By its nature, this form of time perspective goes beyond the individual’s life and focuses rather on the social aspects of problems and phenomena that may exist in the distant future.

The generational time perspective differs from other constructs in the field, such as the transcendental or the transcend time perspective. Transcendentality or transcendence are more related to attitudes towards religion or spiritual development. In turn, The generational perspective has no religious or spiritual aspects and does not focus on one’s personal life after death. Instead of using religious or spiritual issues, the generational time perspective involves an interest in the lives of future generations, curiosity about what the world will look like in several dozen or several hundred years, wondering how science and new technologies will change the lives of future generations, how social processes will then take place and how climate change will change the fate of future generations.

Several constructs have been proposed sharing key aspects with time perspective. The Zimbardo Time Perspective Inventory (ZTPI) is one of the most widely used measures of time perspective^38^. This inventory diagnoses the following dimensions: Past-Negative, Past-Positive, Present-Hedonistic, Present- Fatalistic, and Future. A number of measures relate strictly to the future, for example: Future Time Perspective Scale^39^, the Future Anxiety Scale^40^ or the Consideration of Future Consequences Scale^41^. These scales diagnose different aspects of the future time that is limited by the physical death. In turn, The Transcendental Future Time Perspective Scale (TFTPS) assesses individual attitudes and beliefs about the future immediately after imagined physical death^23^. Also Timoszyk-Tomczyk and Bugajska^24^ proposed tool to examine the time perspective after personal death—The Transcendent Perspective Scale. Both, the transcendental and the transcendent time perspectives diagnose religious or spiritual aspects of one’s personal life after death.

We propose a new tool called the Generational Time Perspective Scale (GTPS), which does not focus on an individual's religious or spiritual beliefs about life after death. Rather than referring to religious or spiritual issues, the generational time perspective demonstrates a concern for the welfare of future generations. That’s the aim of this article is the presentation of a new instrument to diagnose the generational time perspective. In the further part of the article, we present the stages of construction of a tool for diagnosing the Generational Time Perspective.

## Construction of The scale

The inspiration for the construction of the GTPS scale came from a number of sources: our personal experiences and conversations, publications on the future time perspective, and existing diagnostic tools on the subject. Using these sources, we attempted to craft statements about the curiosity of modern people about the living conditions of future generations. In the process of constructing the scale, we also began to consider the affective aspects of the generational time perspective, especially fears and anxieties about life in future generations. The inclusion of the affective aspects of the generational time perspective in the considerations on the generational time perspective was the result of some tragic events in 2022—primarily the war in Ukraine—but also the Covid-19 pandemic and the constantly progressing climate change.

We tried to formulate the questions for the GTPS in a way that the average recipient could understand. The starting point for the questions was the working definition of the generational time perspective: *The generational time perspective is a cognitive-affective representation of the future that relates to the lives of a future generation of people that the current generation of people will not live to see.*

We assigned the initial pool of statements to: (a) the cognitive aspect of GTPS; and (b) the affective aspect of GTPS. We tried to craft statements for each factor in such a way that a person with a particular attitude could disagree or agree with each given statement. For example, some of the statements indicated thinking about the distant future. Some of the statements also described the distant future in negative colours (the affective aspect of the GTPS).

We started the construction of the questionnaire with a pool of 60 items on the phenomenon of generational time perspective. In the next steps, we excluded items that were formulated in a very similar way, were a paraphrase of content already expressed in other items, or contained technical vocabulary. We also excluded statements concerning the consequences of a generational time perspective (e.g. actions for future generations influenced by a generational time perspective).

We sent the initial pool of 25 statements to five experts for evaluation. The experts were psychologists with expertise in time-related issues. They received instructions based upon the scale development standards and recommendations^42^.

Those statements that were judged to be ambiguous, to have the same meaning, or to be too convoluted were eliminated. A total of 17 items were accepted for further analysis. The number of questions in each subscale then proved to be unbalanced: the cognitive aspect of the GTPS contained 9 items, while the affective aspect of the GTPS contained 8 items.

The next stages of the work on the Generational Time Perspective questionnaire are presented below.

## Study 1. Exploratory factor analysis

The first aim of this study was to determine the factor structures of the Generational Time Perspective Questionnaire, and the second aim was to diagnose its reliability.

### Method

#### Participants

The sample consisted of 277 people (Mage = 24.50; SD = 6.55). 196 participants (70.75%) were women and 74 (26.75%) were men. Seven people answered ‘other’ (2.50%). Of the respondents, 50 were university staff (18%). The rest were students (222) (82%).

68 participants (24.50%) lived in villages and 209 (75.50%) participants lived in cities.

#### Procedure

Due to the pandemic, we collected data for the research via the Internet (Google Sheets and a computer application). Google Form is an interactive form whose layout corresponds to the graphical design of the paper equivalent. The subjects filled in the questionnaire directly on the Internet. Their task was to rate each of the statements in the questionnaire and select one of the opinions from a five-point Likert scale. The options were (1)—Definitely disagree to (5)—Definitely agree.

All of the respondents were provided with general comunication about the research objectives and were given a web-based informed consent. The participants were informed that the study was anonymous and that the person could refuse to participate in the study. The email addresses of the respondents were obtained from the university’s email address database. Both students and university staff could participate in the study. The study was approved by the Bioethics Committee of the Institute of Psychology at the University of Szczecin and performed in accordance with the Declaration of Helsinki (KB 27/2022).

### Results

Before proceeding with the factor analysis, we analysed the sampling adequacy (Kaiser–Meyer–Olkin test, KMO) and performed the Bartlett test of sphericity. The KMO measure of sampling adequacy was 0.856, which is a very good result^43^. The Bartlett’s sphericity test: χ^2^(136) = 1787; p < 0.001, indicated that the correlations between the items were high enough to conduct a reliable analysis.

Initial analyses using a parallel analysis suggested the possibility of a two-factor solution (actual λ1 = 5.64, λ2 = 2.55, λ3 = 1.21 vs. λ1 = 1.47, λ2 = 1.37, λ3 = 1.30 from the parallel analysis).

Thus, a possible two-factor solution was investigated, taking into account the loading of their theoretical interpretation. A one-factor solution was also tested. Exploratory factor analysis (EFA) was used to determine the factor structure of the GTPS. Factor solutions were tested using principal axes factoring (PAF) (as it does not depend on multivariate normality assumptions) with Oblimin rotation, as factors may be correlated^44,45^. The individual factor solutions were then assessed for their theoretical and structural interpretation, model coefficients (> 0.40) and eigenvalues (> 0.20)^46^.

Finally, we decided to adopt two factors because this solution best met the assumed criteria.

In the process of refining the scale, we removed three low-load statements for the first factor. (*I imagine what my grandchildren could be like*; *I wonder what might be good for people in the future*; and *I listen to experts talk about the threats to our planet in the future*) and three statements that loaded on the second factor (*I am afraid that the next generations will face much greater threats than those we know about today*, *I feel sorry for people who will live in a few hundred years*, and *I am afraid of what kind of world my children and grandchildren will live in*).

The 11-item version of the Generational Time Perspective Questionnaire was again subjected to exploratory factor analysis using the method of factoring the principal axes. Bartlett’s sphericity test: χ^2^(55) = 1197; p < 0.001. The KMO measure of sampling adequacy was 0.836.

The final version of the Generation Perspective Questionnaire contains 11 items (Table 1).

**Table 1:** Means, deviations, factor loadings, communalities, resources and I-T correlations of the GPC questionnaire (N = 277).

No	ITEMS	M	SD	F1	F2	C		I-T
1	I wonder how people will live in tens/hundreds of years from now	4.00	0.95	0.61		0.41		0.54
2	I feel ashamed that people are destroying the planet and causing problems for future generations	4.19	0.93		0.74	0.55		0.66
3	I wonder what technological inventions will change the world in a few hundred years	3.98	1.11	0.72		0.53		0.65
4	I am worried about what the Earth will look like in the future	3.77	1.06		0.67	0.46		0.63
5	I like to watch films that show life on Earth in the future	3.40	1.32	0.60		0.38		0.53
6	I am afraid of what might happen to life on Earth in the future as a result of climate change	3.96	1.01		0.77	0.60		0.69
7	I like to read articles and books about what life will be like in 200 years’ time	2.92	1.24	0.67		0.45		0.61
8	It upsets me that some people don’t think about what future generations might face	3.86	1.04		0.70	0.50		0.63
9	I think about how people and their relationships with others will change as a result of technological changes	3.88	1.10	0.65		0.42		58
10	I worry about the fate of our planet in the distant future	3.70	1.05		0.77	0.63		0.71
11	I am fascinated by the future of the world	3.66	1.01	0.73		0.54		0.67
	*Cronbach’s α*			0.83	0.85			
	Variance explained [%]			39.20	19.90			

Two factors together explain about 59% of the variance. The first factor explains about 39% of the variance and the second factor explains about 20% of the variance. The factors have satisfactory reliability as measured by Cronbach’s alpha. The reliability of the first factor is Cronbach’s alpha = 0.83, while the reliability of the second factor is Cronbach’s alpha = 0.85.

To summarise, the exploratory factor analysis revealed two factors with satisfactory reliability.

## Study 2. Confirmatory factor analysis

The next stage of the research was to conduct a confirmatory factor analysis. Two models were tested: M1: the first model assumed a one-factor solution; M2 assumed a two-factor solution: the first factor including statements about curiosity about the distant future, the second factor including statements about the affective aspects of the generational time perspective.

We used several indices to evaluate the model on the empirical data: χ^2^(df), comparative fit index (CFI), GFI index, and root mean squared error of approximation (RMSEA). The following criteria indicated a good fit of the model to the empirical data: > 0.90 CFI, > 0.90 GFI, and < 0.09 RMSEA^47^.

### Method

#### Participants

The sample consisted of 228 people (Mage = 22.90; SD = 9.10). 164 (71.90%) women and 60 (26.40%) men took part in the study. Four people marked the answer ‘other’ (1.70%). 153 (67%) people lived in cities and 75 (33%) participants lived in villages.

#### Procedure

As with the exploratory factor analysis data collection, the confirmatory factor analysis data were collected electronically. The subjects filled in the questionnaire directly on the Internet. Each person was informed that the study was anonymous and that participation in the study was voluntary. Consent of the Bioethics Committee of the Institute of Psychology at the University of Szczecin (KB 27/2022).

### Results

The results of the confirmatory factor analysis are shown in Table 2.

**Table 2:** Indices for each model.

Model	χ^2^	p	*df*	GFI	CFI	RMSEA
Model 1	376.213	0.01	44	0.717	0.684	0.156
Model 2	1107.786	0.01	43	0.900	0.911	0.07

The two-factor model proved to be a better fit to the empirical data (χ^2^ = 1107.786; df = 43, p = 0.01; GFI = 0.900; CFI = 0.911; RMSEA = 0.07).

To summarize, the confirmatory factor analysis confirmed the two-factor model solution.

## Study 3: Generational time perspective and similar temporal diagnostic constructs

In Study 3, we looked for possible relationships between the Generational Time Perspective and other instruments for diagnosing orientation in psychological time (convergent validity).

### Method

#### Participants

The sample consisted of 209 people (Mage = 23.30; SD = 7.40). 145 women (71.20%) and 61 men (27.40%) took part in the study, while 3 (1.40%) people marked the answer ‘other’.

#### Procedure

The participants filled in the questionnaire directly on the Internet. Each person to whom we sent the sheet was informed about the purpose of the study, and they were informed that the study was anonymous and that they could refuse to participate in the study. People who took part in the study were also asked to fill in all questionnaires carefully. Consent of the Bioethics Committee of the Institute of Psychology at the University of Szczecin (KB 27/2022).

#### Diagnostic tools

##### Generational Time Perspective Questionnaire

The questionnaire is designed to diagnose generational time perspective in its two aspects: cognitive, related to generational perspective, and affective, related to generational anxiety.

##### ZTPI Time Perspective Questionnaire

The Zimbardo Time Perspective Inventory (ZTPI) by Zimbardo and Boyd^38^ in the Polish adaptation by Aneta Przepiórka, Małgorzata Sobol-Kwapińska and Tomasz Jankowski^48^ is designed to diagnose the four time perspectives: past negative and positive, present hedonistic, and future. In the Polish sample, fatalism was not included in the time perspectives because it probably has a different character. The respondents marked the answers on a scale from 1 (strongly disagree) to 5 (strongly agree). Cronbach’s alpha coefficients ranged from 0.61 for a positive past to 0.83 for a negative past.

##### Transcendental and Transcendent Time Perspective Questionnaire

The Transcendental Time Perspective Questionnaire^24^ includes 18 items. It diagnoses the individual’s focus on life after death and consists of two subscales. The transcendental future refers to ideas that include the time after death, it goes to infinity and includes planning, e.g. meeting loved ones, salvation, etc. It is associated with a belief in the existence of some form of ‘life’ after death. The transcendent future is a holistic approach to the time of life and death, without a strong involvement of the ‘I’. It includes the generational and metaphysical aspects as well as the personal. The subject is asked to respond to the statements on a five-point scale from 1 (I strongly disagree) to 5 (I strongly agree). The reliability of the questionnaire is α = 0.88 for the transcendental future and α = 0.81 for the metaphysical future.

##### Future Anxiety Scale (FAS) Dark Future Scale

*DFS* This scale was developed by Zaleski^49^. It is a one-factor instrument with 25 items. This scale measures future anxiety in a global dimension (e.g., an ecological disaster) as well as in an individual dimension (e.g., a traffic accident). The Cronbach’s alpha coefficient for the scale is 0.92. The author has not established norms, but suggests that the higher the score, the greater the respondent’s fear of the future^40,50^. The study used a shortened version of the Future Anxiety Scale with 5 items^51^. As in the original scale, the respondent is asked to respond to statements on a seven-point scale ranging from 0 (definitely not) to 6 (definitely yes). The short version proved to have good psychometric properties, and Cronbach’s α was 0.88.

### Results

Table 3 shows the relationship between the generational time perspective and other tools for diagnosing psychological time.

**Table 3:** Temporality and generational time perspective.

Temporality	Generational Perspective	Generational Anxiety
Past Positive	− 0.04	0.06
Past Negative	0.08	0.04
Hedonic Present	0.01	0.02
Future	0.14*	0.14*
Transcendent Time Perspective	0.46*	0.25*
Transcendental Time Perspective	0.19*	0.13
Future Anxiety	0.16*	0.40*

*p < 0.05.

Generational perspective correlates positively with future orientation (p < 0.05), transcendental and transcendent time perspective (p < 0.05) and fear of the future (p < 0.05). In turn, generational anxiety correlates positively with the future dimension (p < 0.05), transcendent time perspective (p < 0.05) and fear of the future (p < 0.05).

To summarize, The Generational Time Perspective Questionnaire was demonstrated to have convergent and divergent validity in terms of its correlations with other measures.

## Study 4. Generational Time Perspective and proenvironmental behaviour

The aim of this study was to analyse possible differences in the results of the generational time perspective of people who are involved in activities for the protection of the natural environment and those for whom proenvironmental activities are not important (predictive validity).

### Method

#### Participants

Two groups of people participated in the study. The first group consisted of people for whom the protection of the natural environment is important. Participants in this group engage in behaviours aimed at protecting the natural environment for future generations (Mage = 24.10 SD = 7.70). The control group consisted of people for whom the protection of the natural environment is not important. They do not engage in behaviours aimed at protecting the natural environment for future generations. For them, environmental issues are less important (Mage = 23.10; SD = 3.30).

#### Procedure

Using the university’s database of email addresses, we sent emails to students asking if they were involved in various initiatives such as planting trees, buying ecological products, saving water, taking part in ecological campaigns, etc. Based on the responses received, we distinguished two groups of people: those for whom environmental issues are important and those for whom environmental issues are not important. Consent of the Bioethics Committee of the Institute of Psychology at the University of Szczecin (KB 27/2022).

#### Diagnostic tools

Generational Time Perspective Questionnaire.

### Results

Table 4 shows the differences between activists (those who are involved in pro-environmental activities) and people who are not interested in environmental issues.

**Table 4:** Generational Time Perspective and proenvironmental activity.

	Activists		Controls		t(46)	p
	M	SD	M	SD		
Generational Perspective	3.83	0.85	3.10	0.78	4.14	0.01
Generational Anxiety	4.59	0.54	3.05	0.85	4.59	0.01

People for whom the protection of the natural environment is important, as evidenced by their involvement in proenvironmental behaviour, scored significantly higher on the generational perspective scale (p < 0.01) and the generational anxiety scale (p < 0.01) than people for whom environmental issues are less important.

In summary, the Generational Time Perspective Questionnaire demonstrates predictive validity.

## General discussion

The article presents a proposal for a new tool to diagnose generational time perspective. The questionnaire contains of two subscales: generational perspective and generational anxiety. The generational perspective subscale consists of 6 items. This subscale describes an individual’s focus on the future beyond the life of the current generation. This focus on the future is manifested in an interest in the lives of people in future generations, the development of technology that may change the lives of those generations, or curiosity about interpersonal relationships in future generations. The second subscale, with 5 items, diagnoses negative emotions towards threats that may occur in tens or hundreds of years. These emotions include a fear of future threats as well as anger or shame at what people are doing and how they are behaving now, which may be a potential source of problems for future generations.

The two scales are characterised by validity, reliability and internal consistency. Both the generational perspective subscale and the generational anxiety subscale correlate positively with other assessment tools. Both the Generational Perspective Scale and the Generational Fear Scale correlate with the future dimension of Zimbardo and Boyd’s questionnaire^5,38^, Zaleski’s Fear of the Future scale^49^, and the Transcendent and the Transcendental Time Perspective proposed by Zimbardo and Boyd^5^ and Timoszyk-Tomczak and Bugajska^24^. Note that these correlations are moderate, which may suggest that the generational time perspective scale measures separate phenomena from those diagnosed by these other human time scales.

The generational perspective subscale has the highest correlations with the transcendent perspective subscale^24,25^. Transcendent Time Perspective describes the ability to transcend one’s own ‘I’, a broad view of other people, successive generations, and the sense of oneself as a greater whole. This construct refers directly or indirectly to the theory of self-transcendence proposed by Reed^52^. Reed’s^52^ theory of self-transcendence suggests that self-transcendence involves expanding one’s boundaries in a number of ways: intrapersonally—increasing awareness of one’s philosophy of life, values and dreams; interpersonally—understanding others and one’s environment; temporally—integrating past and future into the present; and transpersonally—connecting with dimensions beyond one’s typical perception of the world.

The positive relationship between the Generational Perspective subscale and the Transcendental Perspective suggests that persons with high scores on generational perspective can expand their spatio-temporal limits. Moreover, they can integrate the past and future into the present.

The generational anxiety subscale correlates relatively strongly with fear of the future^40,53,54^. Fear of the future is defined as a state of fear, anxiety, uncertainty, dread and apprehension of unfavourable changes in a more distant personal future^40^. In turn, generational anxiety is a variety of emotions that arise when thinking about the fate of the next generations on Earth, even a few hundred years from now. The positive correlation between these two types of anxiety may suggest that people with high generational anxiety worry not only about the future of the next generations, but also about their personal future. In continuing research in this area, it would be important to determine the optimal levels of generational anxiety for engagement in environmental activities. It is known that the level of stress can modify performance^55^. In consequence, high levels of generational anxiety can disturb proenvironmental behaviour.

Generational time perspective is weakly correlated with the future in the terms proposed by Zimbardo and his coworkers^51^. The future time perspective, as understood by Zimbardo and Boyd^5^, refers to predicting the consequences of actions and planning. It is a perspective of hopes and threats, but most importantly of opportunities to be exploited in pursuit of one’s ambitions and goals. In turn, the generational time perspective goes beyond the individual future. It focuses on the societal perspective: what might happen in terms of the development of the world and civilization. This is consistent with the results obtained, which show that The Generational Time Perspective Questionnaire distinguishes between people who are involved in various actions aimed at protecting the natural environment for future generations and people who are not interested in doing so. This means that the Generational Time Perspective Questionnaire can predict pro-environmental behaviour, which proves its predictive accuracy.

The validation work on the construction of the questionnaire also has some limitations. One of them is undoubtedly the selection of the research sample. Mostly young people participated in the study. Therefore, it is very difficult to generalise the results obtained. In future research, people from different age groups should be invited to participate.

Although the gender of the respondents was controlled for in the construction of the questionnaire, this variable was not analysed. Previous research, e.g. on the protection of the natural environment, indicates that a person’s gender can influence their involvement in pro-environmental activities (women more often undertake such activities)^56^. It therefore seems necessary to take the variable of gender into account in future studies.

The Time Perspective Questionnaire is a reliable tool, as shown by the Cronbach’s results, but the validation process of the Time Perspective Questionnaire did not include an analysis of the stability of the questionnaire over time, which should be considered a limitation. Future research should measure the stability over time of the questionnaire of Generational Time Perspective.

Future research could also examine the relationship between generational time perspective and various variables of psychological functioning. Do personality traits correlate with generational time perspective? Can generational time perspective be a determinant of personal and social well-being?

The Generational Time Perspective does not include a positive imagination of the future. Perhaps this can be tested in future studies on the Generational Time Perspective Questionnaire.

Furthermore, there is a need to translate and test the scale in English in different cultural contexts.

## Conclusion

The Generational Time Perspective Questionnaire can be a valuable source of scientific reflection in the context of the current age of future threat and opportunity. It appears that the future may be of greater importance to individuals in their daily lives. Therefore, measuring constructs that can diagnose psychological future is important, not only for theory, but also for practice. Such questionnaires as The Generational Time Perspective Questionnaire can assist educators or therapists in effectively delivering diagnoses when they start to work with students or clients on their future. Furthermore, this questionnaire can assist in evaluating the impact of educators' and therapists' work with students and clients. Additionally, the design and development of The Generational Time Perspective can facilitate further advancements in psychology research and practice.

## Data Availability

The datasets used and/or analysed during the current study available from the corresponding author on reasonable request.

## Appendix: GTPS

Below is a series of statements about your attitude to the future, not just the immediate future, but the future including the fate of future generations. Read each statement carefully and decide how much you agree with it using the rating scale below, circling the appropriate number next to each statement.

1. I strongly disagree
2. I rather disagree
3. Difficult to judge
4. I tend to agree
5. I strongly agree

1. I wonder how people will live in tens/hundreds of years from now.
2. I feel ashamed that people are destroying the planet and causing problems for future generations.
3. I wonder what technological inventions will change the world in a few hundred years.
4. I am worried about what the Earth will look like in the future.
5. I like to watch films that show life on Earth in the future.
6. I am afraid of what might happen to life on Earth in the future as a result of climate change.
7. I like to read articles and books about what life will be like in 200 years’ time.
8. It upsets me that some people don’t think about what future generations might face.
9. I think about how people and their relationships with others will change as a result of technological changes.
10. I worry about the fate of our planet in the distant future.
11. I am fascinated by the future of the world.
